# Genome-Wide Identification of the Thaumatin-like Protein Family Genes in *Gossypium barbadense* and Analysis of Their Responses to *Verticillium dahliae* Infection

**DOI:** 10.3390/plants10122647

**Published:** 2021-12-02

**Authors:** Yilin Zhang, Wei Chen, Xiaohui Sang, Ting Wang, Haiyan Gong, Yunlei Zhao, Pei Zhao, Hongmei Wang

**Affiliations:** 1 Zhengzhou Research Base, State Key Laboratory of Cotton Biology, School of Agricultural Sciences, Zhengzhou University, Zhengzhou 450001, China; zhangyilin19971112@163.com (Y.Z.); tingwang2021@126.com (T.W.); 2 State Key Laboratory of Cotton Biology, Institute of Cotton Research of Chinese Academy of Agricultural Sciences, Anyang 455000, China; chenwei01@caas.cn (W.C.); sangxiaohui@caas.cn (X.S.); gonghaiyan@caas.cn (H.G.)

**Keywords:** *GbTLP*, Verticillium wilt, expression patterns

## Abstract

(1) Background: Plants respond to pathogen challenge by activating a defense system involving pathogenesis-related (PR) proteins. The PR-5 family includes thaumatin, thaumatin-like proteins (TLPs), and other related proteins. TLPs play an important role in response to biotic and abiotic stresses. Many TLP-encoding genes have been identified and functionally characterized in the model plant species. (2) Results: We identified a total of 90 *TLP* genes in the *G. barbadense* genome. They were phylogenetically classified into 10 subfamilies and distributed across 19 chromosomes and nine scaffolds. The genes were characterized by examining their exon–intron structures, promoter *cis*-elements, conserved domains, synteny and collinearity, gene family evolution, and gene duplications. Several *TLP* genes were predicted to be targets of miRNAs. Investigation of expression changes of 21 *GbTLP*s in a *G. barbadense* cultivar (Hai7124) resistance to *Verticillium dahliae* revealed 13 *GbTLP*s being upregulated in response to *V. dahliae* infection, suggesting a potential role of these *GbTLP* genes in disease response. (3) Conclusions: The results of this study allow insight into the *GbTLP* gene family, identify *GbTLP* genes responsive to *V. dahliae* infection, and provide candidate genes for future studies of their roles in disease resistance.

## 1. Introduction

Plants are usually exposed to a wide range of pathogens throughout their life cycle, which often triggers complex defense mechanisms, such as upregulation of pathogenesis-related (PR) genes in response to pathogen attacks. Thaumatin-like proteins (TLPs) are a member of PR protein family 5 (PR-5 protein) and are identified by their highly conserved thaumatin domain. Generally, the majority of TLPs contain the following conserved family signature sequence: G-x-{GF}-x-C-x-T-{GA}-D-C-x(1,2)-{GQ}-x(2,3)-C [1,2]. They also contain 16 conserved Cys residues and an REDDD (glutamic acid, arginine and three aspartic acid residues) structure. The eight disulfide bonds in the structure contribute to maintaining the stability of the protein structure, allowing the protein to resist acid, alkali, and protease degradation and heat-induced denaturation [3]. Typical TLPs have a three-dimensional structure which possesses three conserved domains, namely, I, II, and III, and forms a V-shaped acidic cleft on the surface, ensuring the catalytic function of TLPs [3,4].

The TLPs have been shown to exhibit antifungal functions, covering inhibition of fungal enzymes (β-glucanase, xylanase, α-amylase, and trypsin), as well as the ability to lyse fungal cell membranes and spores, and to decrease the viability of germinated spores and induce programmed cell death in fungi [5,6].

TLPs are ubiquitous proteins present in fungi, plants, and animals [7,8]. To date, genome-wide analysis has been used to identify three *TLP* genes in the basidiomycete *Moniliophthora perniciosa*, two *TLP* genes in *Cryptococcus neoformans*, five *TLP* genes in the flour beetle *Tribolium castaneum*, six *TLP* genes in the pea aphid *Acyrthosiphon pisum*, and six *TLP* genes in the nematode *Caenorhabditis elegans* [4]. In contrast, plants have a higher number of *TLP* genes, with 28 in *Arabidopsis thaliana* [2], 31 in *Oryza sativa* [2], 33 in *Vitis vinifera* [9], 28 in *Cucumis melo* [10], and 55 in *Populus trichocarpa* [1], as well as 50, 48, 91, and 90 in *Gossypium raimondii, G. arboreum, G. barbadense*, and *G. hirsutum*, respectively [11].

Transgenic plants overexpressing *TLP* have been shown to enhance resistance against different pathogenic fungi [9,12]. In vitro antifungal activity of leaf extracts from transgenic tobacco and grape expressing exogenous TLPs has been reported to inhibit mycelial growth of *Pythium aphanidermatum*, *Rhizoctonia solani*, *Uncinula necator*, *Botrytis cinerea*, and *Elsinoë ampelina* [9,13,14,15]. To date, endogenous TLPs extracted from banana, peach, and kiwifruit have been proven to be potent antifungal proteins [16,17]. TLPs involved in plant defense responses have been well studied in *O. sativa* and other model systems, with overexpression of an *OsTLP* gene in cassava and banana leading to significantly enhanced resistance against *Colletotrichum gloeosporioides* f. sp. *manihotis* and *Fusarium oxysporum* [18,19]. Transgenic tobacco overexpressing *AdTLP* gene, from the wild peanut *Arachis diogoi*, showed enhanced resistance to the fungal pathogen, *Rhizoctonia solani* [20]. Furthermore, transgenic potato plants overexpressing *CsTLP* from tea (*Camellia sinensis*) exhibited increased resistance against two fungal pathogens [21]. Expression of endogenous *TLP* genes in melon was markedly upregulated in response to *Podosphaera xanthii* infection, and the *TLP*s were also expressed differentially in different tissues and organs [10]. In addition, TLPs in a range of fruits and pollen of apple, cherry, olive, banana, tomato, wheat, and cypress have allergenic activity [22]. Transgenic tobacco plants with constitutively higher expression of the *GbTLP1* showed enhanced resistance against *Verticillium dahliae, Fusarium oxysporum*, and some abiotic stresses including salinity and drought [23]. The *GhTLP19*-silenced cotton exhibited increased sensitivity to *Verticillium dahliae* via increasing the malondialdehyde content and decreasing the catalase content [11].

Globally, cotton is one of the most widely grown commercial crops. It offers not only fibers but also cottonseed oil to the world economy. Nevertheless, cotton productivity has been constrained by a number of biotic and abiotic factors, which cause enormous losses worldwide. A particular yield-limiting factor is the disease Verticillium wilt (VW) caused by *Verticillium dahliae,* which is the most destructive fungal disease affecting cotton production in the world [24,25,26]. *V. dahliae* is a soil-borne fungus, which can survive in soil for many years and has a wide host range of over 400 plant species [27]. *V. dahliae* mainly damages the roots of cotton, causing leaf yellowing, the browning of vascular bundles, the dehiscence of flower buds and bolls, and even the death of the whole plant [28]. At present, the main methods for controlling VW of cotton include resistance breeding and cultural, fungicidal, and biological control. However, in terms of actual production, it is difficult to implement crop rotation due to factors such as land competition between grain and cotton, problems with finding suitable nonhost crops, and its time-consuming and laborious nature. In terms of chemical control, traditional fungicides, which pose a threat to the environment and human health, have been gradually eliminated, while new fungicidal chemicals have an inadequate control spectrum, in addition to retaining potential negative impacts on the environment. Biological control has some limitations, and its control effect is slow and easily limited by climatic and local environmental factors, while the control effect is variable and unstable. Therefore, increasing host resistance is the main focus for controlling VW.

## 2. Results

### 2.1. Identification of TLP Genes in the Sea Island Cotton Genome

Using the hidden Markov model (HMM) profile of the thaumatin domain (PF00314) and the *TLP* sequences from *Arabidopsis* (*n* = 28) and *O. sativa* (31) as queries, a total of 90 predicted *TLP* gene sequences were identified from *G. barbadense*. The 90 *TLP* genes were then sorted according to their chromosomal distribution and relative linear location and named *GbTLP1* to *GbTLP90*, with detailed information listed in Table S1. The 90 *TLP* genes in the sea island cotton genome encoded proteins with different numbers of amino acids. The predicted *TLP* genes encoded proteins ranging in size between 175 (*TLP64*) and 788 (*TLP66*) amino acids, with predicted molecular weights varying between 18.65 and 86.97 kDa. The isoelectric point (pI) ranged from 4.10 to 9.22 (Table S1).

### 2.2. Chromosomal Distribution of GbTLPs and Gene Collinearity Analysis

**Figure 1:**
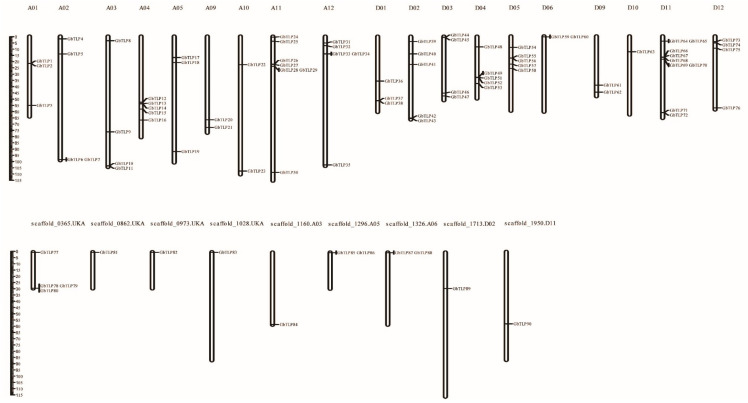
Distribution of *TLP* genes on *G. barbadense* chromosomes (scaffolds). The scale represents megabases (Mb). The chromosome numbers of *G. barbadense* (A01–A12, D01–D12) are indicated above each vertical bar.

Using the genome sequence of sea island cotton as reference, the 90 *TLP* genes identified were mapped onto chromosomes or scaffolds by MapChart. The 90 *GbTLP* genes were distributed across 19 chromosomes and nine scaffolds, with 41 *TLP* genes distributed on 10 Dt chromosomes and 35 *TLP* genes allocated to nine At chromosomes, with the remaining 14 *TLP* genes being located on nine scaffolds (Figure 1). The chromosome with the most genes in the At subgenome is A11, which has seven *TLP* genes, compared with nine *TLP* genes on chromosome D11 from the Dt subgenome. The sea island cotton genome has five chromosomes (chromosomes A02, A03, D03, and D12, and scaffold_0365) each carrying four *TLP* genes, three chromosomes (chromosomes A01, A05, and D01) each carrying three *TLP* genes, and four chromosomes (chromosomes A04, A12, D02, and D05) each possessing five *TLP* genes. Moreover, there are six, seven, and nine *TLP* genes respectively distributed on chromosomes D04, A11, and D11, while the other seven chromosomes (chromosomes D06, D10, scaffold_0862, scaffold_0973, scaffold_1028, scaffold_1160, scaffold_1713, and scaffold_1950) each contain only one *TLP* gene (Figure 1).

**Figure 2:**
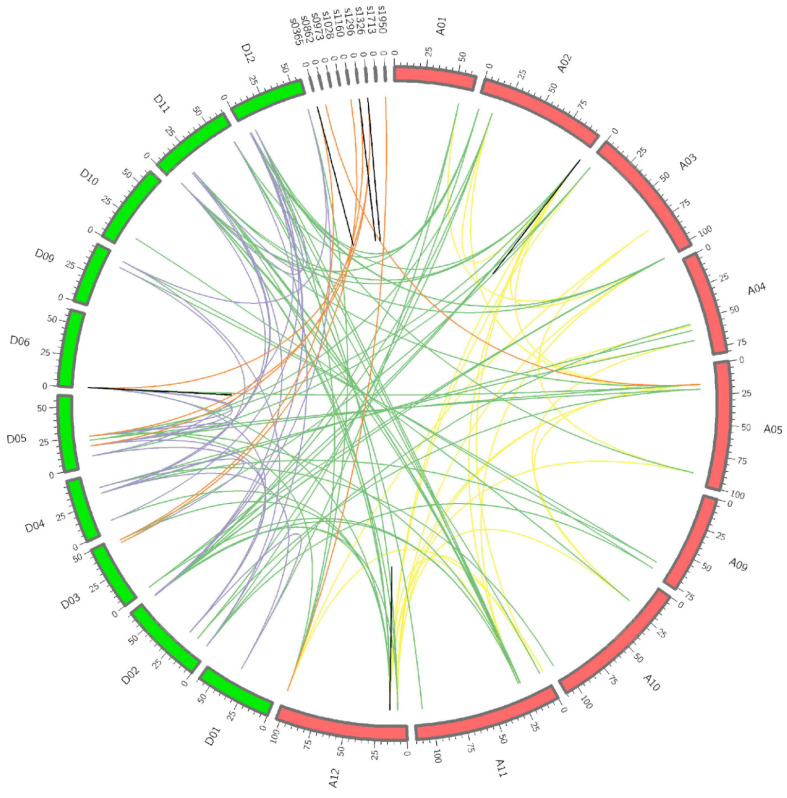
Distribution of segmentally duplicated and tandem duplicated *TLP* genes in the sea island genome. The segmental duplication gene pairs were linked by the lines between chromosomes. The putative *TLP* genes between At and Dt, At and At, Dt and Dt, and chromosomes and scaffolds are connected by green, yellow, purple, and red lines, respectively, while the tandem duplicates are denoted by black lines.

Meanwhile, the mechanism of expansion of the *TLP* gene family was studied by performing a gene duplication event analysis, including tandem duplication and segmental duplication in the cotton species. There were 135 pairs of duplicated *GbTLPs* genes identified in *G. barbadense*. Among them, 13 *TLP* genes (*GbTLP6*, *GbTLP7*, *GbTLP73*, *GbTLP72*, *GbTLP59*, *GbTLP60*, *GbTLP78*, *GbTLP79*, *GbTLP80*, *GbTLP85*, *GbTLP86*, *GbTLP87*, and *GbTLP88*) were clustered in six tandem repeat event regions (namely, chromosomes A02, A12, and D06 and scaffold_0365, scaffold_1296, and scaffold_1326) in the sea island cotton genome, which agreed with the findings of the chromosomal location studies that some genes congregate at certain positions on particular chromosomes (Figure 2). There were two pairs of tandem duplicated genes (*GbTLP78* and *GbTLP79*, *GbTLP79* and *GbTLP80*) within the scaffold region of scaffold_0365, suggesting that *GbTLP78* and *GbTLP80* may have arisen from the same duplication event as *GbTLP79*. In addition, 120 pairs of segmental duplications were found within *GbTLP* in sea island cotton (Figure 2). Notably, all of the segmental duplication gene pairs identified were distributed on different chromosomes in the cotton genome. However, the segmental duplication gene pairs on chromosomes were unevenly distributed; there were 26 pairs of genes duplicated between the Dt subgroups, 23 pairs of genes duplicated between the At subgroups, and only seven pairs of segmental duplicates duplicated between chromosomes and scaffolds. Interestingly, most of the segmental duplication events occurred in 64 pairs of *GbTLP* genes between the At subgroups and Dt subgroups (Figure 2). In addition, of all the segmental duplication events, most occurred between A11 and D11, involving eight pairs of genes.

### 2.3. Phylogenetic and Evolutionary Analysis

**Figure 3:**
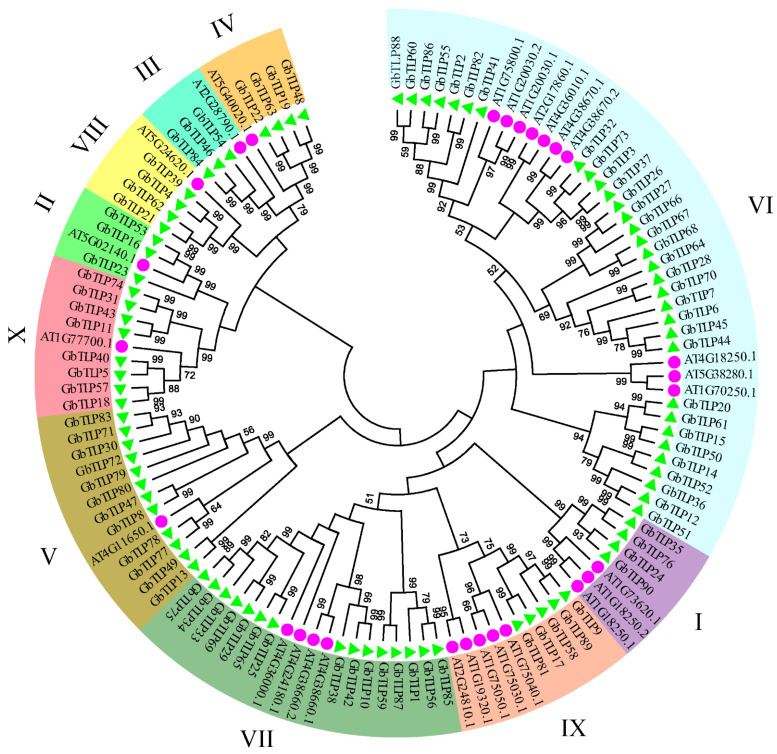
Phylogenetic analysis of TLPs. AT represents *A. thaliana* (purple circle); Gb represents *G. barbadense* (green triangle). The phylogenetic tree was generated using the amino-acid sequences of selected *TLP*s via NJ methods. All sea island cotton *TLP*s, together with their *A. thaliana* counterparts, were classified into 10 groups.

In order to further analyze the evolutionary relationship of *TLP* genes among different species, 118 *TLP* genes were identified from *A. thaliana* (*n* = 28) and *G. barbadense* (*n* = 90), and corresponding phylogenetic trees were constructed (Figure 3). These *TLP* genes could be clearly divided into 10 paraphyletic groups according to their phylogenetic relationship, named groups 1–10 (Figure 3). The results were consistent with previous studies in other plant species, such as *A. thaliana*, *Cucumis melo*, and *P. trichocarpa* [1,7,10]. Each of these subgroups contained at least one *AtTLP* gene, indicating the close relationship between *GbTLPs* and those of other plants. The largest of these groups was group 10, which contained 30 *GbTLP* genes and 10 *AtTLP* genes, whereas the smallest was group 2, which contained three *GbTLP*s and one *AtTLP* (Figure 3). Multiple studies have shown that the gene members in group 5 from other species can respond to pathogenic or environmental stress, and TLPs with antifungal activity tend to have β-1,3-glucanase activity [20,29]. The glucanase activity enables the TLPs to bind to and degrade β-1,3-glucan, the main component of fungal cell walls [30]. This result suggests that the members of this group may play a significant role in plant response to fungal pathogen stress, a finding which is worthy of further research. Analysis of the phylogenetic tree of the *TLP* gene family in group 5 identified 12 *GbTLP* genes, namely, *GbTLP8*, *GbTLP13*, *GbTLP30*, *GbTLP47*, *GbTLP49*, *GbTLP71*, *GbTLP72*, *GbTLP77*, *GbTLP78*, *GbTLP79*, *GbTLP80*, and *GbTLP83*, which are potential candidate genes involved in disease resistance.

Natural selection analysis is widely used to estimate how selection pressure and evolutionary forces affect duplicated genes and their corresponding proteins [31]. In order to investigate the evolutionary origin of the *GbTLP* genes and to further understand the divergence of the sea island cotton *TLP* gene family after polyploidization, estimates of the nonsynonymous (Ka) and synonymous (Ks) nucleotide substitution rates during evolution were calculated, using TBtools, allowing the calculation and analysis of the ratio (Ka/Ks), and using the Ka/Ks ratio to estimate the balance between neutral selection (Ka/Ks = 1), purifying (negative) selection (Ka/Ks < 1), and positive selection (Ka/Ks > 1). The Ka/Ks ratio is a measure used to investigate the mechanisms of evolution of a duplicated gene following its divergence from its ancestral gene [32]. According to the results, 132 of the 135 *GbTLP* paralogous pairs had Ka/Ks ratios <1, suggesting that the *GbTLP* gene family of sea island cotton was mainly affected by high levels of purifying selection. Meanwhile, the remaining three pairs of duplicated genes had a Ka/Ks ratio > 1 (*TLP7* and *TLP6*, *TLP18* and *TLP57*, *TLP30* and *TLP72*), indicating that they evolved under positive selection. In conclusion, the evolution of the *TLP* gene family in sea island cotton was mainly subject to purifying selection, with only a few genes attracting positive selection. At the protein level, the result revealed that the GbTLPs evolved slowly, with conserved structure.

### 2.4. Motif Composition and Gene Structure Analyses

**Figure 4:**
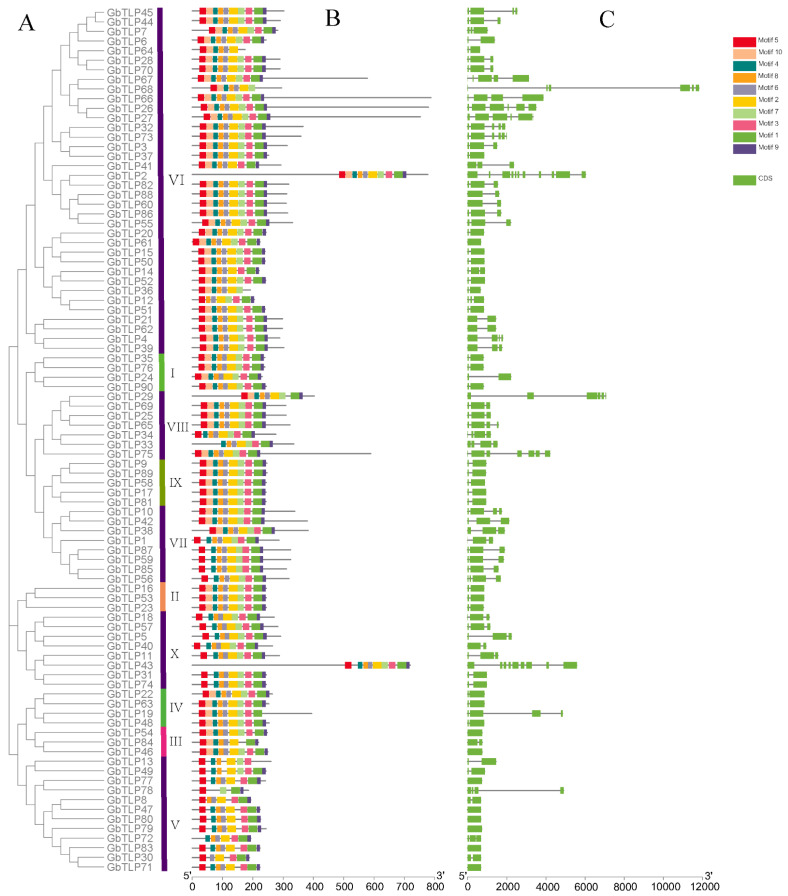
Cluster analysis, gene structure, and domain analysis of sea island cotton *TLP* gene family. (**A**) Phylogenetic tree of *G. barbadense TLP* constructed with MEGA 7.0 by the NJ method. Bootstrap values from 1000 replicates are indicated at each branch. (**B**) Domain compositions of sea island cotton TLP. (**C**) Exon–intron structures of *GbTLP* genes.

Exon–intron structural diversity often plays a key role in the evolution of gene families and can provide additional evidence to support phylogenetic analysis [33]. To gain further insight into the diversification of the *TLP* genes in sea island cotton, the exon-intron organization and conserved motifs were further analyzed (Figure 4). The structural analysis of *GbTLP* genes revealed that the numbers of exons in each gene varied between one and 11. As is well known, different combinations of exons and introns can lead to diverse gene functions. In Figure 4a,c, similar gene structures are shown in the same group, with gene clustering. Compared with the greater variation in the size of the introns, the approximate sizes of the exons were relatively highly conserved among the sea island cotton *TLP* genes (Figure 4c). The protein motifs are highly conserved amino-acid residues that are considered to possibly have functional and/or structural roles in active proteins. In the current study, motif distributions of the 90 thaumatin-like proteins in sea island cotton were analyzed using the MEME program, and 10 conserved motifs, designated motifs 1–10, were identified (Figure 4b). The similar motif arrangements among all 90 *GbTLP*s indicated that the protein structure of the TLPs was conserved in sea island cotton. This is consistent with previous research on the *TLP* gene family, which has been found to be highly conserved, although the functions of most of these conserved motifs remain to be elucidated. Of these, motif 5, motif 2, motif 1, and motif 9 were present in all *GbTLP*s, except for *GbTLP33*, *GbTLP78*, *GbTLP64*, and *GbTLP19*, respectively. Overall, motifs 1, 2, 3, 4, 5, 8, and 9 were detected in all *TLP* members of sea island cotton. In most cases, splice variants of *GbTLP*s showed similar protein sequences with a loss of the motif 2. Despite small differences in motif types among groups, members within the same group tended to exhibit similar motif patterns, such as *GbTLP82*, GbTLP88, *GbTLP60*, and *GbTLP86*, indicating that the functions between them might be extremely similar. It was confirmed that the diversity of *TLP* gene evolution was affected by gene structure and motif patterns.

### 2.5. cis-Element Analysis of GbTLP Promoters

**Figure 5:**
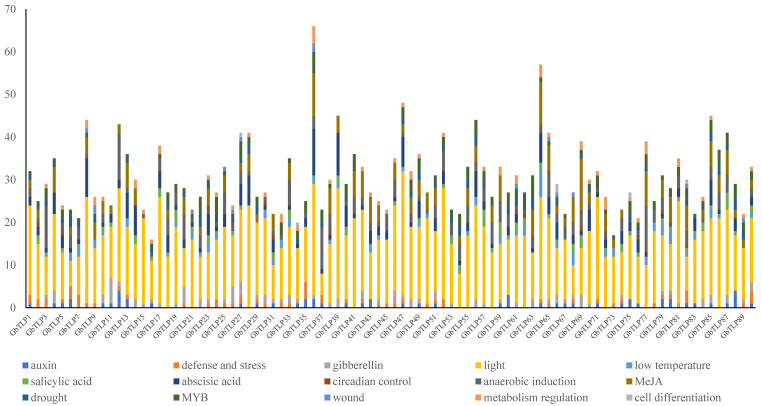
*cis*-Element analysis of putative *GbTLP* promoters. Different *cis*-elements with the same or similar functions are shown in the same color.

The *cis*-acting regulatory element is a specific promoter motif to which an appropriate transcription factor binds in order to regulate gene transcription in plants [34]. We regarded the 2.5 kb genomic sequences upstream of the transcription start site (TSS) of each sea island cotton *TLP* gene as putative promoter regions and used the PlantCARE (http://bioinformatics.psb.ugent.be/webtools/plantcare/) (22 October 2019) tool to identify the presence of *cis*-elements. All 90 *GbTLP* promoters possessed the typical core *cis*-acting elements in promoter regions, including TATA and CAAT boxes. A total of 2753 *cis*-acting elements were observed in the promoter regions of *GbTLP* genes (Figure 5). There were 15 types of *cis*-acting elements, the functions of which included stress response, hormone regulation, cellular development, MYB-binding sites, and metabolic regulation. The results revealed that five types of stress response, namely, anaerobiosis (ARE), drought (MBS), cold stress (LTR), wound stress (WUN-motif), and defense stress (TC-rich repeats), were identified in the *GbTLP* promoter regions. Furthermore, 11 types of hormone regulation elements, namely, ABRE, AuxRR-core, TGA-box, TGA-element, GARE-motif, TATC-box, P-box, CGTAC-motif, TGACG-motif, SARE-element, and TCA-element, which were associated with abscisic acid (ABA), gibberellin (GA), auxin (IAA), methyl jasmonate (MeJA), and salicylic acid (SA) responses, were found in the *GbTLP* promoters. All of them exist in *GbTLP* family members and respond to at least one hormone. Another element, involved in the regulation of cellular development, involves only one of the *cis*-elements, HD-Zip 1, which was associated with cell differentiation, and which is present in five *GbTLP* genes (*GbTLP27*, *GbTLP34*, *GbTLP66*, *GbTLP75*, and *GbTLP84)*. The *cis*-elements of the O_2_-site and circadian were associated with zein metabolism regulation and circadian regulation, respectively, and were present in 27 and 12 *GbTLP* genes, respectively. In addition, many light-responsive elements were present in *GbTLP* promoters. The results showed that different elements in the promoter region of the *GbTLP* gene family may play important roles in regulating plant growth, abiotic stress tolerance, and different hormone responses (Figure 5).

### 2.6. Identification of GbTLPs Targeted by miRNAs

**Figure 6:**
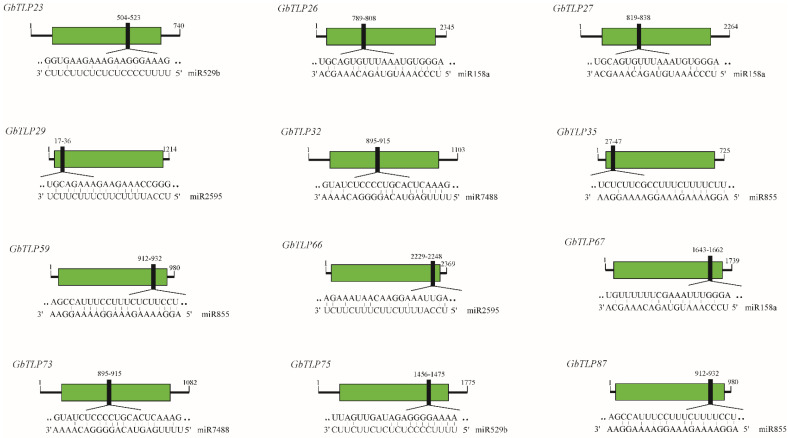
The prediction of targeting regulatory relations between *GbTLP* and miRNAs. Black lines represented ORFs of GbTLPs. The thaumatin domains are represented by boxes filled with green. miRNA complementary sites (black filling) with the nucleotide positions of *GbTLP* cDNAs are highlighted. The RNA sequence of each complementary site from 5′ to 3′ and the predicted miRNA sequence from 3′ to 5′ are shown in the expanded regions.

MicroRNAs (miRNAs) are endogenous noncoding small RNAs, 19–25 nucleotides long, which play important roles in the regulation of plant stress response. They were first discovered in nematodes and are now known to be ubiquitous in eukaryotes [35,36]. miRNAs bind to their target mRNA and initiate the mRNA degradation machinery, leading to mRNA degradation or, in some cases, translational repression. miRNAs play essential roles in metabolism, tissue growth, organ development and differentiation, and apoptosis in plants [37,38,39]. A total of 64 miRNAs associated with VW response were identified from the reported literature. To predict miRNA-mediated post-transcriptional regulation of *GbTLP*s, we searched *GbTLP* coding sequences for target sites of miRNAs, using the psRNATarget server with stricter parameters than the defaults, and predicted 12 *GbTLP*s as targets of five miRNAs (Figure 6). The results showed that *GbTLP35*, *GbTLP59*, and *GbTLP87* were each targeted by miR855, while *GbTLP26*, *GbTLP27*, and *GbTLP67* were each targeted by miR158a; furthermore, miR7488 targeted *GbTLP32* and *GbTLP73*, whereas *GbTLP23* and *GbTLP75* were each targeted by miR529b, and miR2595 targeted *GbTLP29* and *GbTLP66*. All these target sites are located in the thaumatin domain (Figure 6). Our results revealed that the miRNA-mediated post-transcriptional regulation of *TLP* expression might be important for response to VW infection in cotton.

### 2.7. Differential Expression of GbTLP Genes in Response to V. dahliae Infection

**Figure 7:**
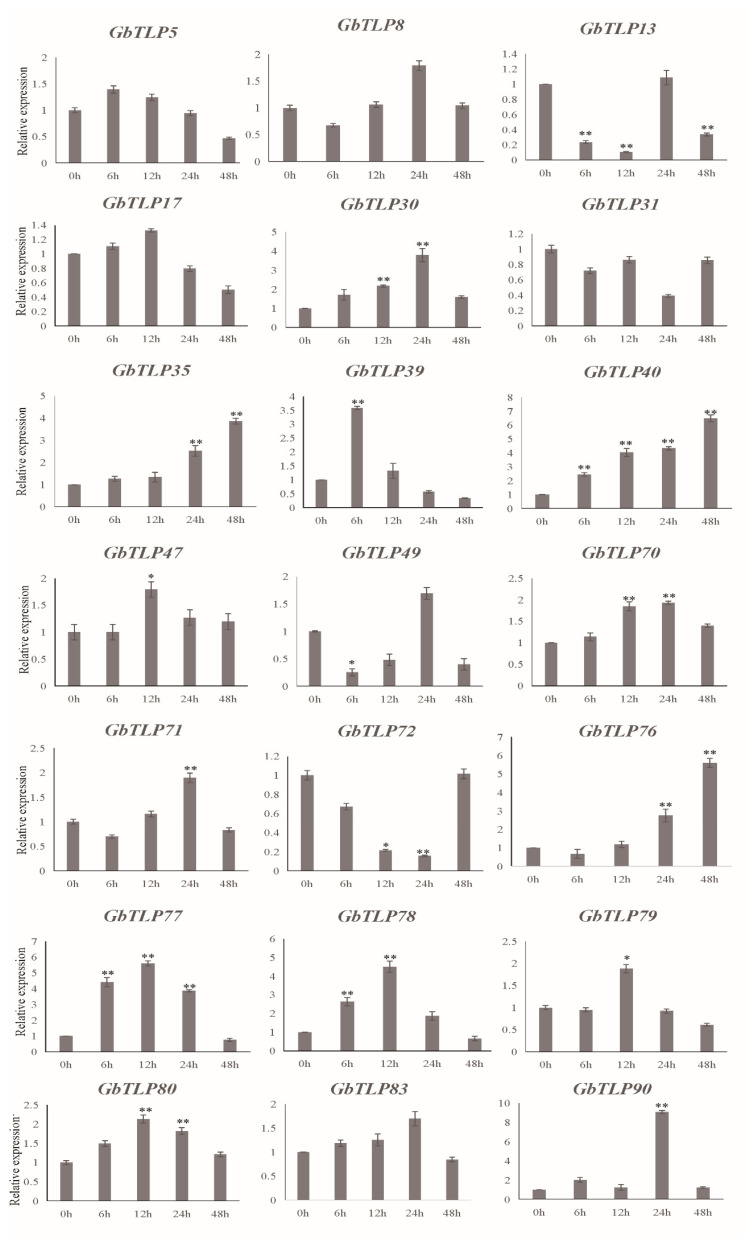
Gene expression of identified *GbTLP* after VW infection. Dark bars represented the relative expression of *TLP* in Hai 7124. Relative expression was calculated using the comparative threshold 2^−ΔΔCT^ method, and values represent averages of three independent biological replicates of three plants each. Error bars were calculated on the basis of three biological replicates using standard deviation; * *p* < 0.05 and ** *p* < 0.01 by Student ’s *t*-test.

To test whether the expression of *GbTLP* genes was influenced by challenge of *V. dahliae*, 21 *GbTLP* members, whose homologs in other plant species were potentially associated with disease resistance according to the previous studies, were analyzed for their responses to *V. dahliae* infection in cotton. The cultivar used in the expression experiments was *G. barbadense* cv. Hai 7124, which is a VW-resistant cultivar. qPCR experiments were performed to analyze the expression patterns of individual *GbTLP* genes in response to challenge by *V. dahliae*. Overall, expression levels of some *GbTLP* genes were significantly up- or downregulated following inoculation with *V. dahliae* compared to the control (0 h) (Figure 7). Expression of 13 of these genes was upregulated at 6 h to 24 h after inoculation with *V. dahliae*, whereas expression of three genes (*GbTLP13*, *GbTLP49*, and *GbTLP72*) was downregulated, with the expression of *GbTLP13*, for example, decreasing after *V. dahlia* infection, reaching the lowest level at 6–12 h, suggesting that these downregulated genes may play crucial biological roles in the VW response in cotton. The remaining genes showed a relatively stable expression at all times in infected and uninfected plants, indicating that they are probably not associated with resistance to VW in cotton. These results suggest that a subset of *GbTLP* genes may play a role in VW resistance in *G. barbadense*.

## 3. Discussion

Thaumatin-like proteins (TLPs) are a large family of plant PR-5 proteins that play a critical role in disease resistance [1]. Genome-wide analyses of *TLP* gene families have been carried out in many species, including plants and animals [7], with some *TLP* genes in plants known to be involved in defense against pathogens [40,41]. The *TLP* gene family has been comprehensively analyzed in *A. thaliana*, rice, grape, wheat, cotton, and melon [1,7,9,10,11]. In the current study, we identified the *TLP* genes in the *G. barbadense* genome, we analyzed the evolutionary relationships of *GbTLP*s, as well as the response to *V. dahliae* infection of a subset of *GbTLP* genes.

### 3.1. Expansion of the GbTLP Gene Family in Sea Island Cotton

In this study, we identified 90 putative *TLP* genes (*GbTLP1* to *GbTLP90*) from the genome of sea island cotton. In a previous study, 91 TLP genes were identified mainly because different reference genomes and identification procedures including the selection of E-value were selected [11].

On the one hand, the results of gene duplication analysis suggested a mass of gene duplication events in the cotton genome. *GbTLP* genes were found to cluster into six tandem duplication event regions on six chromosomes (scaffolds) (Figure 2). Moreover, several tandem duplicated genes were classified into the same subfamily, implying that they may have originated from recent gene duplication events. In addition, the results of collinearity analysis indicated that segmental duplication was the main approach during duplication of the *GbTLP* gene family. These results clearly revealed that tandem duplication and segmental duplication were the major factors leading to the expansion of the *TLP* gene family in sea island cotton.

Natural selection analysis is widely used to estimate how natural pressures and evolutionary forces affect duplicated genes and the corresponding proteins encoded [41]. Selective pressure includes positive selection, purifying selection, and neutral selection. In sea island cotton, evolution of the majority of *TLP* genes was shown to be driven by purifying selection, whereas only three pairs of duplicated genes were brought about by positive selection, including the pairs *GbTLP6* and *GbTLP7*, *GbTLP18* and *GbTLP57*, and *GbTLP30* and *GbTLP72*, indicating that six genes evolved by natural selection for mutations beneficial to the organism. A study on poplar *TLP* genes suggested that four TLP kinases and 10 TLPs underwent positive selection [2], indicating that the poplar TLPs may have experienced diversified natural selection processes, similar to that which occurred in the current study with the *TLP*s in sea island cotton. Most *TLP* sequences evolved under the influence of purifying (negative) selection pressure, whereas on only a few sites in *TLP* sequences did positive selection operate during the evolutionary process, paralleling results from studies on the evolution of *TLP*s in *A. thaliana*, rice, and maize [42].

In addition, the causes of gene expansion may be due to different mechanisms of evolution. Liu et al. studied 118 *TLP* gene sequences, which were divided into nine groups, with the main genes belonging to the five groups IV, VI, VII, VIII, and IX, and they considered that the *TLP* genes originated about 1.0 × 10^9^ years ago in the common ancestor of plants, animals, and fungi [2]. Compared with the TLPs in *A. thaliana*, which consist of 10 evolutionary groups, the TLPs in sea island cotton were also divided into 10 branches (Figure 3). It has been found that the TLPs of other species in evolutionary group 5 can respond to pathogenic and environmental stresses. For example, *AT4G11650*, also known as *ATOSM34*, encodes a protein which participates in the signaling pathway of *A. thaliana* to respond to pathogenic microorganisms and to salt stress [43].

### 3.2. The Putative Regulatory Mechanisms of GbTLP Gene Expression

A number of research studies have shown that TLPs are localized in the extracellular space, enhancing plant tolerance to various stresses [20,44]. *TLP* gene expression can be regulated by a variety of signals, such as SA, MeJA, abscisic acid, injury, UV, osmotic stress, and invasion by bacteria, fungi, viruses, and other organisms [45]. In the *GbTLP* promoter regions, we found a number of stress-responsive *cis*-elements, such as ARE, MBS, LTR, WUN-motif, and TC-rich repeats, which are responsive to biotic and abiotic stresses (Figure 5). We also found several phytohormone-associated regulatory elements in the *GbTLP* promoters, which indicated that the *GbTLP* gene family probably participates in phytohormone signaling pathways. Specifically, we noted the presence of ABRE, TGA/AuxRR-core, and GARE motif elements, which were associated with ABA, gibberellin, and auxin responses, respectively. According to reports, ABA accumulates under stress conditions, plays an important role in plant stress response and tolerance, and may coordinate reactive oxygen species (ROS) signaling pathways. For instance, tobacco TLPs induce yeast cell apoptosis through the accumulation of ROS, mediated by the RAS2/cAMP pathway [46], showing that the plant TLPs exhibit antimicrobial activity and may be related to the accumulation of ABA. Thus, multiple *cis*-regulated elements upstream of the gene coding sequence are essential for gene-specific expression. *TLP* gene expression is regulated by specific *cis*-elements in the promoter region. Plants respond to abiotic and biotic stresses in a variety of ways, including through the interaction between molecular and cellular changes to improve tolerance to abiotic stress and the control of related metabolic process mechanisms. These mechanisms involve multiple systems and are based on the synergistic effect of cascades of signal transduction networks, involving multiple genes, such as the response patterns between gene expression and hormones in plants. For example, gCC-boxes in the osmotic protein of tobacco gene promoter region are necessary for ethylene response [47]. However, evidence on the upstream regulation of *GbTLP*s and on the downstream factors regulated by *GbTLP*s at different levels is lacking. Furthermore, the relationship between the plant innate immune system and the mechanism regulating expression of *GbTLP* genes, as well as the non-coding miRNA-mediated stress tolerance in cotton, needs additional investigation.

### 3.3. Expression Patterns of GbTLPs Responding to Verticillium Wilt Treatment

In recent years, the antifungal activity of TLPs has been studied in detail. More than 20 TLPs from animals, fungi, and plants have been found to have antifungal activity. The TLPs in plants inhibited both pathogenic and nonpathogenic fungi by lysing fungal spores, as well as inhibiting spore germination and reducing the growth of young hyphae [8,15,48]. Most studies have shown that transgenic plants expressing recombinant *TLP*s delayed the development of a number of fungal diseases and enhanced the resistance of plants to pathogenic fungi [9,12]. The expression profiles of *GbTLP* genes in response to VW infection revealed their potential functions in response to challenge by pathogenic fungi and helped in understanding their biological roles. In this study, we found that the expression of 13 *TLP* genes including *GbTLP30*, *GbTLP35*, *GbTLP39*, *GbTLP40*, *GbTLP47*, *GbTLP70*, *GbTLP71*, *GbTLP76*, *GbTLP77*, *GbTLP78*, *GbTLP779*, *GbTLP80*, and *GbTLP90*, was significantly increased in VW-resistant cotton cv. Hai7124 following VW infection, suggesting that expression of these genes may correlate positively with cotton resistance to VW. These results were consistent with previous studies and supported the finding that many *TLP* genes exhibit antifungal activity [11]. For example, a recombinant ClTLP27 protein isolated from watermelon drastically reduced the mycelial growth of several fungal phytopathogens [12]. In cherry tomato, recombinant protein LePR5 was found to exhibit antifungal activity against *Cryptococcus laurentii* [49]. However, some TLPs did not exhibit antifungal activity. Two osmotin-like proteins from latex lacked anti-fungal activity against several fungi phytopathogens [50]. Elderberry (*Sambucus nigra*) and barley *TLP* (*HvPR5b*, *Pr22-1*, and *Pr22-2*) were also found to exhibit no antifungal activity [51,52,53,54].

In the present work, three downregulated *GbTLP* genes, namely, *GbTLP13*, *GbTLP49*, and *GbTLP72*, were also identified in response to VW attack. The expressions of these genes were severely repressed, indicating that they may be involved in distinct physiological events rather than in host defense. Although numerous studies regarding the biological roles of *TLP*s have shown that proteins exhibited antifungal activities, detailed information on their functions is still uncertain when it comes to new species. More information about the TLPs reported here is needed to obtain a better understanding of the role played by these proteins in sea island cotton.

## 4. Materials and Methods

### 4.1. Identification and Annotation of Sea Island Cotton TLP Genes

The genomic database of *G. barbadense* (NAU) was downloaded from the Cotton Functional Genomics Database (CottonFGD) (https://cottonfgd.org/) (10 March 2019). The HMM profile of the thaumatin domain (PF00314) was downloaded from the Pfam website (http://pfam.xfam.org/) (25 March 2019) and was used as the query to identify all possible thaumatin-like sequences with HMMER software (http://hmmer.org) (25 March 2019). Furthermore, the conserved *TLP* domain of each candidate sequence was confirmed by Pfam. In the sea island cotton genome, we identified 90 *TLP* gene family members and obtained the details of individual *TLP* genes in sea island cotton, including genomic position, protein length, molecular weight (kDa), isoelectric point, and exon–intron structure information through the CottonFGD (https://cottonfgd.org/) (30 March 2019).

### 4.2. Chromosomal Location Analysis

The physical chromosome locations of all *TLP* genes were acquired from the genome sequence databases. According to the gene coordinates in the genome annotation, the chromosomal distribution of *TLP* genes in sea island cotton genome was drafted using Mapchart 2.3 software.

### 4.3. Synteny and Collinearity Analysis

We used BLAST (version 2.6.0) for the pairwise comparison of the filtered TLP sets of *G. barbadense*, and then MCscanX was employed to identify homologous regions, while syntenic blocks and duplicate gene classifications were evaluated using Circos-0.69. Default parameters were used in all the steps.

### 4.4. Phylogenetic and Evolutionary Analysis

We used two species to study the evolutionary relationship between the *TLP* genes, namely, sea island cotton and *A. thaliana*. The sequence data of *A. thaliana* were collected from the National Center for Biotechnology Information (NCBI) (http://www.ncbi.nlm.nih.gov/protein/) (5 May 2019). Multiple alignments for all of the available and predicted TLP full-length protein sequences were performed using Clustal X2 with manual adjustment, where appropriate, for the alignment of the *TLP* domain. A phylogenetic tree was constructed using the neighbor-joining (NJ) method of MEGA 7.0.

### 4.5. Gene Structure and Conserved Motif Analysis of the TLP Gene Family

The genetic structure of *GbTLP* genes, including the exon–intron structure, was analyzed and mapped using the Gene Structure Display Server (GSDS 2.0). The conserved domain of *TLP* was analyzed using the online software MEME (http://meme-suite.org/) (15 July 2019), which was used to analyze the protein sequences of the *G. barbadense*-designated GbTLPs. The maximum number of discovered modules was set to five, with the other parameters being tacit values.

### 4.6. Prediction of Regulatory cis-Elements in the Promoter of GbTLPs

The genomic sequences at 2.5 kb upstream of the transcription start site (TSS) of each *GbTLP* gene were extracted from the genome files of *G. barbadense* cv. Hai 7124. The PlantCARE server (http://bioinformatics.psb.ugent.be/webtools/plantcare/html/) (22 October 2019) was used to predict the transcriptional response elements of *GbTLP* gene promoters [55]. We obtained cotton miRNA sequences from the published literature [56,57]. *GbTLP* genes targeted by miRNAs were predicted by searching coding sequences (CDS) regions for sequences complementary to the cotton miRNAs using the psRNATarget server (http://plantgrn.noble.org/psRNATarget/home) (18 November 2019) [58] with default parameters, except for maximum expectation (E) = 3.0 and maximum unpaired energy (UPE) = 20.0.

### 4.7. Plant Materials and Treatments

To study the responses of selected *GbTLPs* to challenge by *V. dahliae* in sea island cotton, a seed from *G. barbadense* cultivar cv. Hai 7124 was planted in potting soil at 25 °C in a culture room with a 16 h light/8 h dark cycle. Young plants at the four-true-leaf stage were inoculated with *Vd991*, a highly aggressive defoliating strain of *V. dahlia*, using the root dip inoculation method; the strain was cultured in complete medium at 25 °C for 5 days, with the concentration of conidia for inoculation adjusted to 5 × 10^6^ conidia/mL. The seedling leaves were harvested with three biological replicates at five time intervals (0, 6, 12, 24, and 48 h) after *Vd991* inoculation. Each replicate consisted of five seedlings, which were immediately frozen in liquid nitrogen after harvest and stored at −80 °C until used for total RNA extraction. Total RNA from these samples was isolated using RNAprep Pure Plant Kit (polysaccharide- and polyphenolic-rich, DP441) (Tiangen, Beijing, China). The first-strand cDNA fragment was synthesized from total RNA using PrimeScript^®^RT Reagent Kit (Takara, Kusatsu, Japan). Then, the cDNA templates were subjected to 10-fold dilution and used for qPCR. The quality and concentrations of the isolated RNA samples were determined by 1% agarose gel electrophoresis and spectrophotometrically with a NanoDrop 2000 spectrophotometer (Thermo Fisher Scientific, Wilmington, DE, USA), respectively. Reverse transcription PCR was carried out using HiScript^®^ II Q RT SuperMix for qPCR with gDNA wiper (R223) (Vazyme, Nanjing, China) on samples at the five timepoints.

### 4.8. Quantitative Real-Time PCR Analysis

Gene-specific primers used for qPCR amplification are listed in Table S2 and were designed using Primer Premier 5.0 by avoiding conserved regions within the members of the *TLP* gene family. Transcript levels were determined using a 7500 Real-Time PCR system (Applied Biosystems) and SYBR^®^ Premix Ex Taq™ II (Tli RNaseH Plus) (Takara, Japan), with three technical replicates of each biological sample. The 20 μL reaction volume contained 10 μL of SYBR Premix Ex Taq II (Tli RNaseH Plus) (2×), 0.8 μL each of the PCR forward and reverse primers (10 μM), 0.4 μL of ROX Reference Dye II (50×), 2 μL of cDNA, and 6 μL of ddH_2_O. The thermal cycling conditions were as follows: an initial denaturation step of 30 s at 95 °C, followed by 40 cycles of 5 s at 95 °C for denaturation, 34 s at 60 °C for annealing, and a melting curve step at 95 °C for 15 s, 60 °C for 1 min, and 95 °C for 15 s. *GbActin* was used as an endogenous reference gene, and a melting curve analysis was performed. Samples at 0 h were used as calibrators in qPCR analyses. Cycle threshold (C_t_) values were used for the relative quantification of the input target number. The relative expression levels of genes were calculated using the 2^−ΔΔCT^ method, to normalize the variance among samples. For statistical analysis, standardization of gene expression data from three independent biological replicates per experiment was performed, and all reactions were performed with three technical replicates. The results were statistically analyzed using a Student’s *t*-test at *p* < 0.05.

## 5. Conclusions

We identified 90 putative *GbTLP* genes in the sea island cotton genome, which are distributed across 19 chromosomes and nine scaffolds and might be derived from polyploidization or segmental duplications. Analysis of the expression patterns of selected *GbTLP* genes in a VW-resistant cotton accession identified *GbTLPs* responsive to *V. dahliae* infection, implying their potential role in resistance to VW. Analysis of the promoter sequences revealed *cis*-acting elements associated with phytohormone signaling and stress response, with different members harboring distinct types and numbers, suggesting that individual members of the *GbTLP* gene family might be differentially regulated at the transcriptional level. Several *GbTLPs* contain target sites of miRNAs that have been previously shown to be associated with VW response, implying potential miRNA-mediated post-transcriptional regulation of *GbTLPs* in disease resistance. Collectively, our study provides a comprehensive analysis of the expression, regulation, and evolution of the *GbTLP* gene family, and it lays the foundation for further cloning and functional characterization of the *GbTLP* genes using a reverse genetics strategy.

## Data Availability

Not applicable.

## Supplementary Materials

The following are available online at https://www.mdpi.com/article/10.3390/plants10122647/s1, Table S1. The *TLP* gene family in *Gossypium barbadense*; Table S2. Primer sequences used in the study.

## Abbreviations

PR	Pathogenesis-related
TLP	Thaumatin-like protein
VD	Verticillium dahlia
pI	isoelectric point
